# The impact of active stakeholder involvement on recruitment, retention and engagement of schools, children and their families in the cluster randomised controlled trial of the Healthy Lifestyles Programme (HeLP): a school-based intervention to prevent obesity

**DOI:** 10.1186/s13063-017-2122-1

**Published:** 2017-08-14

**Authors:** J. Lloyd, C. McHugh, J. Minton, H. Eke, K. Wyatt

**Affiliations:** University of Exeter Medical School, University of Exeter, St Luke’s Campus, Heavitree Road, Exeter, EX1 2 LU UK

**Keywords:** Recruitment, Retention, Engagement, Stakeholder involvement, Cluster RCT, Child, School, Obesity prevention, Complex intervention

## Abstract

**Background:**

Recruitment and retention of participants is crucial for statistical power and internal and external validity and participant engagement is essential for behaviour change. However, many school-based interventions focus on programme content rather than the building of supportive relationships with all participants and tend to employ specific standalone strategies, such as incentives, to improve retention. We believe that actively involving stakeholders in both intervention and trial design improves recruitment and retention and increases the chances of creating an effective intervention.

**Methods:**

The Healthy Lifestyles Programme, HeLP (an obesity prevention programme for children 9–10 years old) was developed using intervention mapping and involved extensive stakeholder involvement in both the design of the trial and the intervention to ensure that: (i) delivery methods were suitably engaging, (ii) deliverers had the necessary skills and qualities to build relationships and (iii) the intervention dovetailed with the National Curriculum. HeLP was a year-long intervention consisting of 4 multi-component phases using a range of delivery methods. We recruited 1324 children from 32 schools from the South West of England to a cluster-randomised controlled trial to determine the effectiveness of HeLP in preventing obesity. The primary outcome was change in body mass index standard deviation score (BMI SDS) at 24 months post randomisation. Secondary outcomes included additional anthropometric and behavioural (physical activity and diet) measures at 18 and 24 months.

**Results:**

Anthropometric and behavioural measures were taken in 99%, 96% and 94% of children at baseline, 18 and 24 months, respectively, with no differential follow up between the control and intervention groups at each time point. All children participated in the programme and 92% of children and 77% of parents across the socio-economic spectrum were considered to have actively engaged with HeLP.

**Conclusions:**

We attribute our excellent retention and engagement results to the high level of stakeholder involvement in both trial and intervention design, the building of relationships using appropriate personnel and creative delivery methods that are accessible to children and their families across the social spectrum.

**Trial registration:**

International Standard Randomised Controlled Trials Register, ISRCTN15811706. Registered on 1 May 2012.

## Background

### Why consider recruitment and retention?

Successful recruitment is paramount for high-quality randomised controlled trials (RCTs). Under-recruiting in relation to the trial target leads to a reduction in statistical power (i.e. an insufficient sample size to avoid a type II error [1]), which can negatively affect the reliability of the trial results. In addition, prolonged recruitment can increase trial costs [2], affect the timely dissemination of the findings into practice [3] and impact relationships built with those who have already committed to the study. However, once participants have been recruited, the next challenge is to retain them throughout the duration of the trial. Some attrition is always expected in applied research; however, when this rate exceeds 20%, bias is expected in the results [4].

Evidence suggests that in industrialised countries low socio-economic status (SES) groups are at higher risk of becoming overweight/obese [5]. It is important, therefore, that behavioural trials to treat and prevent obesity are able to recruit, retain and engage children and their families across the social spectrum and report on any differences by socio-economic group. Recent reviews of both obesity prevention and management trials have highlighted low participation, and high rates of dropout and loss to follow up [6–9]; however, few studies discuss the problems experienced with recruiting and retaining children and families or the strategies and/or approaches employed that were helpful [10, 11].

Recent research shows a number of promising strategies to improve recruitment into clinical trials such as the use of direct rather than passive targeting of participants, opt-out approaches for consent and incentives and reminders [12, 13]. In health-related community-based studies involving schools, child care centres and other youth-related organisations, the most promising strategies include building trustful relationships between researchers and study partners (head teachers, child care centre directors), parents and children; having project champions; optimising consent and follow-up procedures; offering incentives to study partners, children and parents; minimising participant burden and designing feasible studies with cohesive research teams [14].

These are all useful strategies for other researchers to use, however, *how* to successfully build and maintain relationships with trial participants such that they are engaged with the research and happy to participate in the study overall are rarely reported [15]. We believe that building such relationships goes beyond the use of standalone strategies and requires undertaking a “relational approach” to both the design of the trial and the intervention, which begins with the co-creation of the research question and evaluative designs to be used and is reflected on throughout the research process.

### The Healthy Lifestyles Programme (HeLP)

**Fig. 1:**
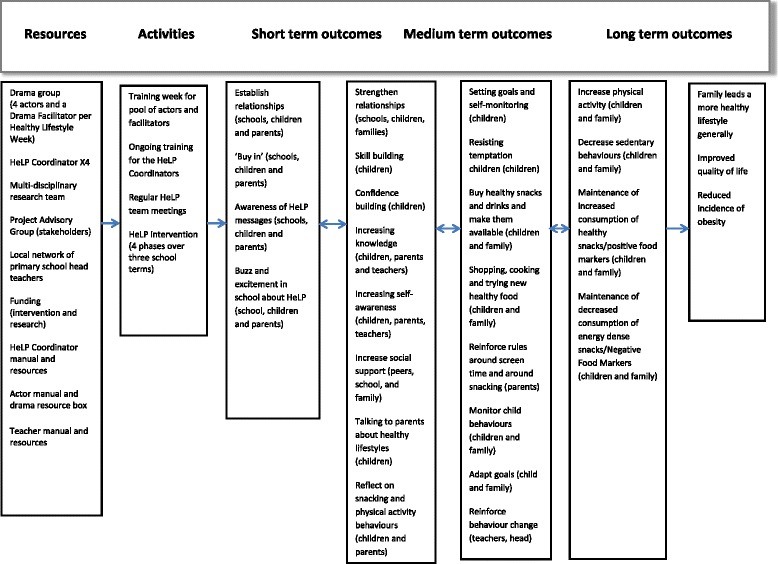
The logic model for the Healthy Lifestyles Programme (*HeLP*)

The HeLP was a multi-component, school-based obesity prevention intervention, which was developed using an intervention mapping approach [16] with extensive stakeholder consultation [17]. The programme consists of 4 phases, delivered over three school terms to all year-5 children (9–10 years old) within a school. Each school had one key contact person called the HeLP Coordinator (HC) who liaised with school staff and parents. They were also involved in delivering aspects of the intervention and liaising with other delivery personnel. The overall aim of HeLP was to deliver a general healthy lifestyle message, encouraging a healthy energy balance with a focus on three specific behaviours relating to energy intake and energy expenditure; decreasing the consumption of sweetened fizzy drinks; increasing the ratio of healthy to unhealthy snacks consumed and reducing screen-based activities. Figure 1 shows the HeLP intervention logic model, which attempts to visually represent the theoretical underpinning of the intervention, its content, the process by which it was assumed to work, the context in which it was delivered and the predicted outcomes.

During the intervention, there were six invitations for parents and carers to come into the school and take part in the programme. These include the parent assembly and observation of the two activity workshops in phase 1, observation of work in progress in the final two drama sessions of the Healthy Lifestyle Week in phase 2 and the forum theatre assembly in phase 3. Full details of intervention phases, components, behaviour change techniques and delivery personnel have previously been published [18].

Four HCs were recruited for the study period and were allocated eight schools each (four intervention and four control schools). The role of the HC was to oversee the collection of measurements in both intervention and control schools and the delivery of HeLP in their four intervention schools. HeLP Coordinators were also responsible for delivering many components of the programme and were seen as central to building relationships with schools, children and families and supporting teachers throughout the study. All HCs were graduates, with experience of working with children and families either in a research capacity or as teachers.

This paper presents how we embedded meaningful stakeholder involvement in both the design of the trial and the intervention in order to develop successful strategies in the HeLP cluster RCT to maximise recruitment, uptake of the intervention, completeness of follow-up data and the engagement of schools, children and their families across the socio-economic spectrum.

## Methods

### Trial design

**Fig. 2:**
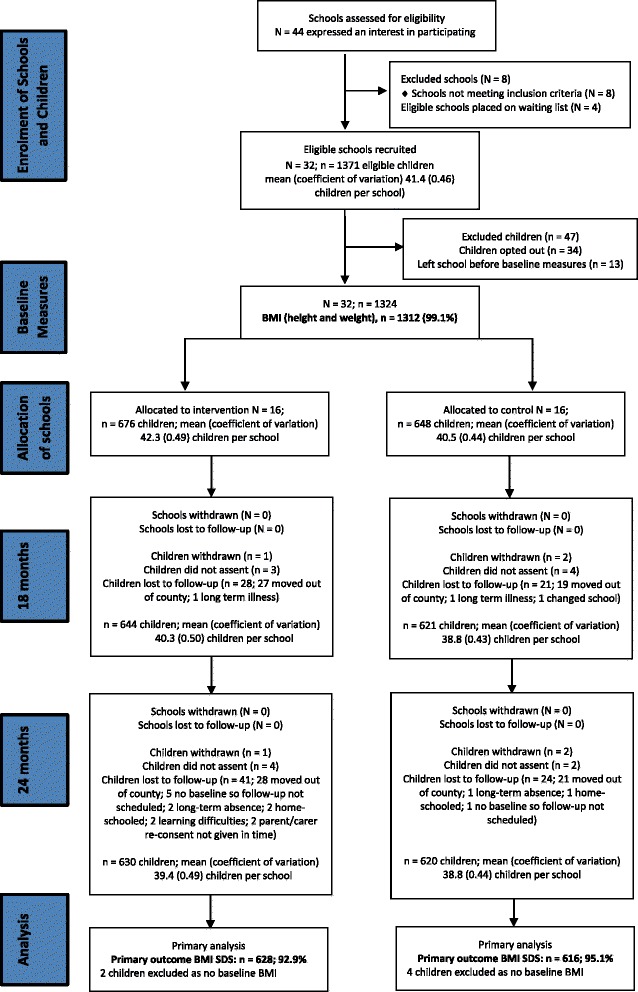
Trial profile showing school and child recruitment and retention. *N* refers to the number of schools (clusters) and *n* refers to the number of children (individual participants). Two schools that had been allocated to cohort 2 withdrew whilst waiting to commence the trial and so were subsequently replaced with two of the four schools on the waiting list, prior to cohort 2 commencing. All schools that started the trial remained within the trial and so all the randomised clusters are present at baseline and at each follow-up point. The percentage in *brackets* for the proportion of children with data at both baseline and follow up is calculated from the total number of recruited children in the schools at baseline. Not all children with a follow-up measure necessarily had a corresponding baseline measure (or vice versa) due to different children being absent on the day of the main and additional assessments for each of the time points, and/or due to children leaving or moving between schools. In all the analyses, children were analysed in the group (intervention or control) to which the school they were enrolled in at baseline was randomized

The cluster-randomised controlled trial of the HeLP assessed the effectiveness and cost-effectiveness of HeLP in preventing children being overweight or obese [19]. The primary outcome was change in body mass index standard deviation scores (BMI SDS) at 24 months post randomisation. Based on the trial power calculations [19] and assuming a 20% loss to follow up, we needed to recruit 28 schools of varying sizes with a total of at least 952 children to ensure that we had 24-month outcome data from 762 children for the analysis. We decided to recruit 32 schools to ensure we had a minimum of 28 schools completing the trial, each with an estimated average of 35 year-5 children. To ensure that we had a representative sample of schools in terms of socio-economic status, we aimed to have half of our recruited schools with ≥19% of pupils eligible for free school meals, which represented the national average at the start of the trial. In July 2012, following the recruitment period, half of the schools were randomised to receive HeLP and half acted as control schools following stratification by the proportion of children eligible for free school meals (<19%, ≥ 19%) and class size (one year-5 class, >1 year-5 class). For practical reasons half of the schools (eight intervention and eight control) entered the study in 2012 (cohort 1), and the other half in 2013 (cohort 2). Children were recruited in September 2012 for cohort 1 and in September 2013 for cohort 2, using an opt-out process. Children were measured at baseline (before group allocation was revealed to schools and research staff) and then at 12, 18 and 24 months post baseline (see Fig. 2).

### Trial outcome measures

#### Anthropometric measures (taken at baseline, 18 and 24 months)

Height was measured using a SECA stadiometer (Hamburg, Germany), recorded to an accuracy of 1 mm. Weight was measured using the Tanita Body Composition Analyser SC-330 (U.K. Ltd., Middlesex, UK). Weight was recorded to within 0.1 kg and children were asked to take off their shoes and socks. BMI was calculated and converted to centiles using the software package LMS, developed by Cole [20]. Percent body fat (%BF) was estimated from leg-to-leg bioelectric impedance analysis (Tanita Body Composition Analyser SC-330) and converted to centiles using the LMS software [21]. Waist circumference was measured using a non-elastic flexible tape measure, 4 cm above the umbilicus. At each data collection time point, children had the option to decline measurement if they so wished.

#### Behavioural measures (taken at baseline, 12 months (My Lifestyle Questionnaire (MLQ) only) and 18 months)

Physical activity was assessed using the GENEActiv accelerometer. One randomly selected class from each school was asked to wear a GENEActiv accelerometer [22] a device worn like a watch around the wrist during waking and sleeping hours over 8 consecutive days, allowing for one day of familiarisation. Information packs were sent directly home to parents 1 week prior to the children coming home with the watches so that they were aware of all the procedures discussed with the children on the day of “hook up”. This included instructions regarding how to refit the watches should they be removed, a colourful reminder sheet to put up in the house and letters for children to distribute to coaches to explain the study and the importance of keeping the watches fitted during sporting activity if this was appropriate. On the day of “hook up” the HeLP Coordinator spoke to 10 children at a time about the watches and ensured that all understood how to comply with procedures. Watches were fitted to the child’s non dominant hand and children were encouraged to wear them for 24 hours a day.

Food intake was assessed using the adapted version of the validated Food Intake Questionnaire (FIQ) [23]. The FIQ asks children about the food and beverages they consumed the previous day and allows an estimation of the number of healthy and unhealthy food and drink items consumed per day. Children complete the FIQ twice in order to obtain a weekday and weekend food intake. The HC led the two lessons required for the children to complete the questionnaires. Children were arranged in literacy groups to ensure that help could be given as efficiently as possible. Another researcher and the class teacher and teaching assistant provided support when required.

The My Lifestyle Questionnaire (MLQ) assessed knowledge, individual motivations and cognitions, parental behaviours, child use of change techniques and specific behaviours that mediate levels of physical activity and food intake in children [19]. The HCs collected the MLQ data during a dedicated lesson and read the questions from the front of the class, with the children completing the questionnaire at the same time and in silence. Clarifications were given for specific questions. Children who required additional support completed the questionnaire in a smaller group outside of the classroom with an additional researcher.

The Index of Multiple Deprivation (IMD) score was assigned to the lower super output area of each pupil as determined by their postcode [24]. IMD scores were grouped into quartiles in order to assess any differential effect of engagement by socio-economic status.

### Assessment of uptake

Participation by children and their families (for parental engagement events), was assessed using registers of attendance with percentages of children or parents attending each component and/or phase calculated.

### Assessment of engagement (assessed for intervention particpants only, during delivery of the intervention)

All children had a one-to-one discussion with the HC in phase 3 about their goals. It was during this interaction that the HC gave each child an engagement score between 0 and 3 (0 = disinterested/unaware goals needed to be set; 1 = reluctant/needs a lot of prompting; 2 = enthusiastic and happy to chat about goals and how they will achieve them; 3 = very enthusiastic, has discussed them at home and has clear strategies for achieving them). The HC had worked closely with the class for 10 months at this time, thus had the insight to carry out an accurate assessment. These scores were then dichotomised to create two groups (≤1 = less engaged children and >1 = engaged children).

Parental engagement was measured using two sources of data; attendance at one or more parent events and/or signature on the goal setting sheet. A score between 0 and 2 was given to each parent (0 = did not attend/did not sign; 1 = attended or signed the goal setting sheet, but not both; 2 = attended one or more events and signed the sheet). As with child engagement these scores were dichotomised to create two groups (≥1 = engaged, <1 = not engaged).

School engagement was assessed using three scores based on the HC’s interaction with the head teacher, the year 5 teacher(s) and the support staff. A score between 0 and 3 was given to each group (0 = unengaged/uncooperative; 1 = supportive; 2 = enthusiastic and supportive; 3 = very enthusiastic and used HeLP in other aspects of teaching/school activities). These scores were aggregated and then dichotomised into two groups (0–3 = less engaged school and 4–9 = engaged school).

### Strategies to optimise recruitment of schools and children and completeness of follow up data: stakeholder involvement in trial design

From the outset we worked with a group of teachers, head teachers, parents and children from the early piloting of HeLP [25] who became our Project Advisory Group (PAG). Membership of this group has increased as we progressed though the piloting phases. Meetings were held when required at times that were convenient to the group (usually 4.00–6.00 p.m.) and all expenses were paid including cover for teaching staff if this was necessary. The composition of the group depended upon the focus of the meeting. Our PAG members advised us on what was feasible and acceptable when taking behavioural and anthropometric measures from 9–10-year-old children and how to communicate with parents about the research process so that they: (a) would receive the information, (b) understand it and (c) feel they were able to engage with the researchers if they had any concerns or queries. In addition, it was important for us to understand how to recruit schools and engage teachers. The head teacher in our PAG suggested we recruit schools via a regional network of primary school heads, the Devon Association of Primary Heads (DAPH) during one of their quarterly briefing sessions, and a teacher involved in the exploratory trial [26] offered to talk to heads about her experiences of being involved in the programme during this session. We were also advised to recruit schools via the Academic Learning Partnerships (ALP), which consists of clusters of approximately eight schools in a particular location. Head teachers of these partnerships meet regularly, thus providing an opportunity to target schools to increase the chances of achieving a representative sample. We presented the study at the two locality DAPH meetings and invited schools to sign up there and then to participate. We also attended two ALPs to discuss the study at one of their meetings to recruit further schools in order to meet our deprivation criteria.

Teachers and parents from our PAG were invited to be partners on our research bids and both our funded exploratory [26] and definitive trial [19] had a year-5 teacher and a parent as a co-applicant.

Table 1 details the main strategies used to engage schools and parents with the study overall to ensure that we were able to recruit sufficient schools and children across the socio-economic spectrum and, crucially, follow up as many children as possible at both 18 and 24 months. These strategies were based on the advice of our PAG and lessons learnt during the piloting phases [25, 27]. Strategies have been grouped under three headings.

**Table 1:** Strategies to engage schools and parents with the study

Written communication
• The HC kept regular email contact with teachers, keeping them informed throughout the trial.
• Easy to read information leaflets were created for parents to inform them about the collection of data a week prior to measures being taken (for the 12, 18 and 24 month time points)
• Any parent letters and/or flyers relating to the trial were sent home in book bags and were put into envelopes with labels saying “to the parent/carer of XX”, as parents reported that this looked more official, thus they were more likely to receive and read them.
• An information leaflet was created for non-trial schools that received trial children during the course of the study, so that they were aware of the need to see these children at follow up.
• In schools with a high proportion of English as an additional language, all parental correspondence was translated.
• Large print/coloured versions of letters and information flyers were created for parents with visual impairments.
Verbal communication
• The HC met the teachers some time before the parent information packs were distributed (September 2012 in cohort 1 and September 2013 in cohort 2 schools) to discuss the details of the study. A teacher flyer was created for them to take away.
• The HC was available in the playground to speak to parents on several occasions during the intervention and during the period when children were being recruited (October 2012/2013).
• The HC made contact with the year-6 transition member of staff (at the end of the summer term of year 6, before the children moved on to their allocated secondary schools) to ensure secondary schools were aware of the study, and an information leaflet was created for the transition lead in all secondary schools.
School and parent support
• Envelopes, stamps and address labels were given to administrative staff when letters needed to be sent directly home to parents. The HC also offered to help complete this task.
• The contact details of the HC assigned to the school were on all correspondence to parents and a poster with their picture and contact details was displayed by the school reception desk.
• The HC was available to meet with parents if they had any concerns and/or queries about the trial and/or the intervention for the duration of the study.

### Strategies to optimise engagement with the intervention: stakeholder involvement in intervention design and delivery

Our PAG not only advised us on how best to recruit and engage participants so that they remained in the trial, but they also co-created the intervention with us as our research partners. They provided invaluable feedback on possible intervention activities and delivery methods ensuring they were acceptable and feasible for schools, children and their families. It was important that any intervention we developed did not widen existing health inequalities and had the potential to engage children and their families from across the socio-economic spectrum. We wanted to encourage children to identify with, and take ownership of, the healthy lifestyle messages and take them home to their parents and discuss them with their peers [18].

Our PAG also highlighted the importance of quality delivery by personnel who were able to engage school staff, children and their families. Teachers in our group commented that having one key contact person would help build relationships and that this person should have the necessary skills and competencies to understand the busy lives of teachers and parents. They believed that a background in working with children would be really important in understanding how children behave and respond. From these discussions the role of the HC was developed and the parents on our PAG helped recruit the HCs for the cluster RCT in addition to providing critical feedback during practise delivery of the parent assemblies. Table 2 summarises engagement strategies used within the intervention and throughout delivery.

**Table 2:** Strategies to promote engagement with the intervention

Intervention design
• Phase 1 of the intervention focussed on creating a receptive context, essential for the successful delivery of subsequent components.
• The use of interactive drama as a delivery method, built around a framework of four characters (*Football Freddie, Snacky Sam, Active Amy and Disorganised Duncan*)*.* The attributes of these characters related to the key messages of the programme.
• Children chose which character they most resembled, and then worked with that actor to help the character learn to change their behaviour.
• Children co-created scenes with the characters and actors.
• Learning was based on the relationship between fiction and reality, allowing children to role-play real-life situations.
• The HC was the key contact for schools, children and families, providing support and building relationships.
• Intervention activities fitted in with the National Curriculum at Key Stage 2, by covering many key objectives for science, mathematics, literacy and personal, social and health education (PSHE).
• Children set personalised goals at home with their parents, followed up with a one-to-one discussion with the HC.
Intervention delivery
• In the main, trained personnel (outside the school) were used for delivery (sports/dance groups, actors, the HC).
• Teachers were required to deliver the PSHE lessons and actively observe the interactive drama sessions during the Healthy Lifestyles Week to promote engagement with the programme.
• Delivery of the drama sessions was dynamic and fun and involved a number of behaviour change techniques such as role play, problem solving, role modelling and identification of barriers.
• All components were responsive to the needs of every child in the class.
• Components could be adapted slightly to better fit the context of the school, whilst still remaining true to the programme.
• Each component of the intervention was manualised.
• The building of relationships was at the heart of intervention delivery.

## Results

### Recruitment of schools and children

Within a 4-month period, 36 schools signed up to participate following the two DAPH briefing events and 8 schools signed up following the two ALP meetings, giving a total of 44 primary schools, of which 36 were eligible (having at least one year-5 class of 20 or more children). There were 32 schools that we purposely sampled to ensure that trial schools represented a range of sizes (one to three year-5 classes), locations (urban and rural) and deprivation (5–53% eligible for free school meals). The remaining four schools were asked if they were prepared to go on a “wait list” in case a cohort-2 school dropped out during the waiting year. All four schools agreed. There were a total of 52 classes across the 32 schools and 1371 children were eligible to participate. There were 34 children who opted out prior to baseline measures (17 in cohort 1 and 17 in cohort 2) and 13 children had left school before baseline measures were taken, giving a total of 1324 children (see Fig. 2).

### Completeness of baseline and follow-up data

At baseline, we achieved 99% or more completeness of data collection for all anthropometric and behavioural measures at baseline (see Table 3). At 12-month follow up (immediately after delivery of the final phase of the intervention) MLQ data were collected from 95.7% (630/658) of children in cohort 1 and 97.3% of children (648/666) in cohort 2 (96.5% in total).

**Table 3:**
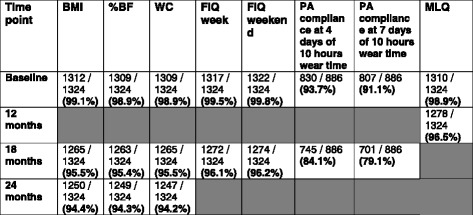
Completeness of anthropometric and behavioural data

The grey boxes indicate that measures were not collected at this time point

The 18-month follow up occurred in May/June when the children were in year 6, following their Standardised Assessment Tests. The measures taken at this time point were anthropometric, food intake and physical activity. Anthropometric data were collected by assessors blinded to group allocation. To obtain 24-month measures, children were tracked to their respective secondary schools (*n* = 60 schools) where only anthropometric measures were taken, by assessors blinded to group allocation (see Fig. 2). Table 3 shows the completeness of data at each time point for anthropometric and behavioural measures, respectively.

### Intervention uptake

Across both cohorts 676 children were randomised to receive the intervention. Child attendance registers were kept for each component in each phase: 52% (353/676) of children had family attending at least one parent invitation. Table 4 shows the percentage of children participating in each phase of the HeLP intervention and the total percentage of children receiving key components i.e. providing children with the information, motivation and behavioural skills in the drama workshops and leading them to take action through the use of personalised goal setting and support. Intervention uptake was exceptionally high for all phases and across both cohorts.

**Table 4:** Uptake of HeLP across the four phases for each cohort

	Phase 1	Phase 2	Phase 3	Phase 4	Percentage of children receiving 4 drama sessions and the goal setting^a^ delivered in the spirit of HeLP^b^
Number of components	5	10	2	4	5
Cohort 1 (*n* = 254)	91.2%	94.1%	91.1%	92.1%	93.7%
Cohort 2 (*n* = 422)	94.7%	93.7%	92.5%	91.4%	92.7%
Total	93.4%	93.9%	92.0%	91.6%	93.0%

*HeLP* Healthy Lifestyles Programme

^a^Dose of HeLP deemed to be essential for behaviour change

^b^Enthusiastic delivery, open body language, responsive to child/school needs and clear and friendly communication

### Child, parent and school engagement

There were 96% of children (652/676) who set goals with the HC in phase 3 and of these, 63% (411/652) had parental support (indicated by a parent signature on the goal setting sheet and/or written comments regarding how the parent would support the child in achieving their goals). In total 92% (602/652) of children were deemed to be engaged with the HeLP programme with little difference between genders (91% of boys and 94% of girls). There was no difference in IMD rank spread between the engaged and less engaged children, suggesting that HeLP was able to engage across the social spectrum.

There were 77% of parents (520/676) deemed to be engaged with HeLP. There was no difference in IMD rank spread between the two groups, suggesting that HeLP was also able to engage parents across the social spectrum. Out of the 16 intervention schools, only three were categorised as less engaged. Reasons for this included administrative and teaching challenges due to external school assessment pressures and staffing issues due to the absence of the head and/or teachers, whereby the drama workshops were viewed as an opportunity to free up teachers to do “other things”.

## Discussion

Poor recruitment, attrition and lack of engagement of study participants in research studies, especially RCTs, are common problems [28–30] and can lead to bias in the findings [31]. Furthermore, “differential attrition” (i.e. differences between follow up in the control and intervention groups) is regarded as a major threat to internal validity of a study [32]. The average rate of attrition in trials is 18% in the intervention group and 17% in the control group, but there is indication of a slightly higher amount of attrition on average in the intervention conditions of trials of change in health behaviour [33]. Most school-based randomised controlled trials for obesity-related behaviour change have reported attrition rates of between 30% and 20% for objective measures taken at follow up that is greater than 12 months [6–9].

The HeLP trial has an attrition rate of only 4% and 6% at 18 and 24 month follow-up, with no differences by cohort or between the control and intervention groups at either of the time points. Although follow up for anthropometric measures in obesity prevention trials is relatively high (70–80%), compliance for objective measures of physical activity in RCTs has been typically lower. In addition, many studies use a less stringent compliance threshold for inclusion in the analysis, which has implications for external validity [34–36]. The 84% compliance with accelerometry at the stringent 4-day threshold of at least 10 hours wear time at 18 months follow up for the HeLP trial provides the most comprehensive of any available for children of this age group. We also have 79% of children with the full 7 days of data, which will allow us to better understand the associations between children’s daily and total physical activity and anthropometric outcomes.

We attribute our high levels of recruitment, retention and engagement to our meaningful stakeholder involvement in both the design of the trial and the intervention and a focus first and foremost on relationship building using appropriate personal and innovative and creative delivery methods that are accessible to children and their families across the socio-economic spectrum. This participative approach, which involved children, their parents, teachers and head teachers, ensured that schools felt confident that participation would not significantly affect their workload, parents and children were adequately informed about the trial and their options to participate, meant that all trial outcome measures were taken in ways that were acceptable to children. There was no discussion of weight or size, and children (and control schools) were rewarded for their participation. Our approach is consistent with the Chief Medical Officer’s view that researchers “… should work with children and adolescents to input to the design of clinical studies … to facilitate (their) increased participation in trials.” [37].

Qualitative data (which will be presented in detail in a future paper) showed that children across the socio-economic spectrum engaged with, and enjoyed HeLP, as they were actively involved in creating scenes and helping the characters, which motivated them to take the messages on board and to engage their family in making changes [27]. Our delivery personnel were carefully selected and trained to ensure they had the necessary skills and competencies to build relationships, with stakeholders being involved in the recruitment process and in running delivery practice sessions. In pilot work, teachers and parents commented that having one key contact person, who was both accessible and approachable, was crucial in feeling supported throughout the duration of the programme [18].

Several authors have written about the issues concerned with recruiting children into trials, although the issues raised are largely concerned with treatment trials where individual children are being recruited [13, 38, 39]. HeLP was an obesity prevention programme and hence was aimed at all 9–10-year-olds regardless of weight status. Whilst other studies of lifestyle interventions for primary school children have had similar levels of inclusion and compliance with baseline measures, follow-up measures have been less complete [36, 40, 41]. Involving schools, parents and children from across the socio-economic spectrum in supporting recruitment processes, assessment procedures and in helping us build relationships with secondary schools, all played a central role in our recruitment, uptake, retention and engagement.

The strengths of the present study are that the trial had a robust design with an extensive process evaluation involving mixed methods. Unlike many other trials, we report data, not only on recruitment, uptake and retention, but also on the engagement of schools, children and their parents, which we believe to be a prerequisite to behaviour change and crucial to achieving high levels of retention in randomised controlled trials. We do, however, acknowledge that the engagement measure was somewhat subjective, although we do have qualitative data (to be reported elsewhere) to support the engagement findings presented in this paper.

Future research in school-based trials of health-related programmes need to ensure that stakeholders are actively involved in the research process from the inception of the research question through to the dissemination of the findings. Not only will this ensure that prevention programmes are feasible and acceptable to the context in which they are delivered but it will also ensure that engagement with the programme across the socio-economic spectrum is as high as possible. High levels of recruitment and retention are essential to demonstrate the representative nature of the participating populations and hence permit conclusions to be drawn to aid future research.

## Conclusion

We attribute our excellent retention and engagement results to the high level of stakeholder involvement in both trial and intervention design, the building of relationships using appropriate personnel and creative delivery methods that are accessible to children and their families across the social spectrum. Our recruitment, retention and engagement results will allow us to make definitive conclusions about the outcome of the trial.

## Abbreviations

ALP: Academic Learning Partnerships
BMI: Body mass index
FIQ: Food Intake Questionnaire
HC: HeLP Coordinator
HeLP: Healthy Lifestyles Programme
IMD: Index of Multiple Deprivation
MLQ: My Lifestyle Questionnaire
NIHR: National Institute for Health Research
OFSTED: Office for Standards in Education
PAG: Project Advisory Group
RCT: Randomised controlled trial
SDS: Standard deviation scores
SES: Socio-economic status
